# Polymeric Scaffolds: Design, Processing, and Biomedical Application

**DOI:** 10.3390/ijms22094552

**Published:** 2021-04-27

**Authors:** Piotr Dobrzyński, Elżbieta Pamuła

**Affiliations:** 1 Centre of Polymer and Carbon Materials, Polish Academy of Sciences, ul. Curie-Skłodowskiej 34, 41-800 Zabrze, Poland; 2 Faculty of Science and Technology, Jan Długosz University in Częstochowa, ul. Armii Krajowej 13/15, 42-200 Częestochowa, Poland; 3 Department of Biomaterials and Composites, Faculty of Materials Science and Ceramics, AGH University of Science and Technology, Al. Mickiewicza 30, 30-059 Kraków, Poland

Tissue engineering is a fascinating and multidisciplinary field of science. In general, it concerns the implementation of scaffolds, cells and biologically active molecules so as to create functional living tissues. Tissue engineering provides an opportunity to treat diseases that cannot be effectively cured with now-existing therapies. In many cases, such a modern approach can extend the life expectancy of patients and improve their comfort and well-being.

The combination of biomedical technologies and novel synthetic biomaterials can support the regeneration and repair of tissues or damaged organs. Possibly, in the future, entire body parts will be created thanks to the achievements of tissue engineering. The further development of biocompatible and biodegradable structures supporting human cells, i.e., scaffolds or artificial matrices, is one of the major goals in the field of biological substitutes intended to restore or improve tissue functions. There is also a cell-free approach, which can be equally effective. For instance, carefully designed bioactive scaffolds are capable of attracting progenitor cells, which populate the scaffold, and thus, restore the damaged tissues. This is why, the design of scaffolds, the synthesis of advanced polymeric biomaterials endowed with application-specific properties and innovative polymer-processing techniques play a key role in the development of novel therapies.

This book is a collection of two reviews and twelve research articles that present the latest trends in polymer scaffold design, their relevance and possible future developments. Due to the vastness of the subject matter, the book includes just a few examples of the most prospective advances in the synthesis and application of polymeric and polymer-based scaffolds for tissue engineering and regenerative medicine.

The first article by Haugen et al. provides an overview of injectable hydrogel scaffold materials of natural and synthetic origin in dental tissue engineering and regeneration [1]. The second review article focuses on bioresorbable polymeric scaffolds for cardiovascular applications, e.g., bioresorbable stents, vascular grafts and cardiac patches [2]. Both articles address a very important approach in modern regenerative medicine and tissue engineering—the development of less invasive therapies that use polymeric biomaterials and composites with enhanced properties.

Most of the articles deal with biomimetic composite scaffolds for bone tissue engineering and regeneration. The scaffolds can be made of resorbable aliphatic polyesters and inorganic mineral phase [3,4,5], hydrogels of natural origin, e.g., collagen/chitosan [6], chitosan [7], gelatin [8] or hydrogels of synthetic origin based on modified poly(vinyl alcohol) [9,10]. Kopeć et al. [11] explore a polydopamine coating on the polyurethane surface in order to improve the mechanical properties of the obtained biomaterials, to reduce their hydrophobicity and to enhance the cell adhesion. Kostopoulos et al. [12] present the recent advances in the composite polyetherimide/hydroxyapatite/graphene scaffold manufacturing via electrospinning. A very promising method of producing scaffolds mimicking the extracellular matrix with bioactive functional motifs is proposed by Pugliese and Gelain [13], who designed self-assembled peptides exhibiting self-healing properties. The last article shows an original approach to the production of hepatic organoids based on pullan and dextran matrices [14]. Organoid technology is more and more often used as an in vitro tool for testing novel drugs. It also appears to have great potential in tissue engineering and personalized medicine.

## Data Availability

Not applicable.
